# Phylogenetic and similarity analysis of HTLV-1 isolates from HIV-coinfected patients from the South and Southeast regions of Brazil

**DOI:** 10.1186/1742-4690-8-S1-A68

**Published:** 2011-06-06

**Authors:** Mariana C Magri, Helena K Morimoto, João L P Ferreira, Rosangela Rodrigues, Luis F M Brigido, Adele Caterino-de-Araujo

**Affiliations:** 1 Instituto Adolfo Lutz, São Paulo, SP, Brazil; 2 Faculdade de Ciências Farmacêuticas - USP, São Paulo, SP, Brazil; 3 Universidade Estadual de Londrina, Paraná, Brazil

## Background

HTLV-1 is endemic in Brazil (0.07-15.9%) and HIV/HTLV-1-coinfection has been detected, mostly in the North and Northeast regions. This study characterized HTLV-1 isolates from HIV-coinfected patients from Southern and Southeastern Brazil.

## Material and methods

DNA from 17 HIV/HTLV-1-co-infected patients; 5 from the South and 12 from Southeast were amplified by nested-PCR (env and LTR) and sequenced. HTLV subtyping was performed by NCBI-Genotyping and REGA-Subtyping tools websites and by phylogenetic methods using Paup4 and MEGA4.

## Results

Env sequences (705-bp) from 15 isolates and LTR sequences (731-bp) from 17 isolates were obtained. Molecular analysis of env and LTR sequences disclosed respectively, nucleotide similarities of 99.5% and 98.8% within sequences, 99% and 97.4% with ATK, and 91.6% and 90.3% with Mel5. Four sequences that showed the highest similarities with ATK clustered separately in the trees (p≤0.001 for env and LTR, bootstrap=96% for LTR); they had C386T and T594C nucleotide substitutions in LTR sequences. All except these four sequences had mutations T5637G, T5730C, A6120G in env and T500A, A549G, G676A, T766C, T767G in LTR sequences. Six sequences presented the amino acid change V1981I and clustered together in the env tree. All isolates belonged to Cosmopolitan HTLV-1a subtype.

## Conclusions

These data show that Cosmopolitan HTLV-1a subtype is also frequent in HIV-coinfected patients from Southern and Southeastern Brazil, suggesting spreading of HTLV-1 in the country. The mutations observed should be monitored in the context of molecular epidemiology.

